# 

**Published:** 1872-02

**Authors:** 


### ARTICLE VI.

### *Eastern Ohio Dental Association.*

BY DR. D. B. MCLAIN.

The second semi-annual meeting of the Eastern Ohio Dental Association, will be held at Alliance, in Masonic Hall, commencing at 10 o'clock A. M., on Tuesday, February 27th, 1872. Session will continue one day, to which all members of the profession are cordially invited. Interesting clinics will be given by competent operators.

*Subjects for Discussion.*—Anæsthesia. The Gums and their Diseases, and when is Extraction Indicated.

*Essayists.*—J. M. Porter, of Massillon; J. H. Siddall, of Canton.

Major Kerr, of the Arlington House, will entertain members and visitors, at very reasonable rates during the convention.

J. C. Whinery, *President.*

D. B. McLain, *Secretary.*

